# Climate complexity in the migratory cycle of *Ammodramus bairdii*

**DOI:** 10.1371/journal.pone.0202678

**Published:** 2018-08-27

**Authors:** Alexander Peña-Peniche, Irene Ruvalcaba-Ortega, Octavio Rojas-Soto

**Affiliations:** 1 Red de Biología Evolutiva, Laboratorio de Bioclimatología, Instituto de Ecología, A.C., El Haya, Xalapa, Veracruz, México; 2 Universidad Autónoma de Nuevo León UANL, Facultad de Ciencias Biológicas, Laboratorio de Biología de la Conservación y Desarrollo Sustentable, Ciudad Universitaria, San Nicolás de los Garza, Nuevo León, México; Universita degli Studi di Napoli Federico II, ITALY

## Abstract

One way to understand the ecology of bird migration is to analyze how birds use their ecological niche during their annual cycle. *Ammodramus bairdii* is a grassland specialist sparrow that breeds in southern Canada and the northern U.S.A. and winters in the Chihuahuan Desert. A continuous and alarming decrease of its populations has been observed over the last 50 years, and studying its seasonal distribution and associated climatic niches could help improve strategies for its conservation. We analyzed the temporal use of its Grinnellian niche (GN) -set of environmental conditions under which a species can establish and persist; in this case the climatic attributes-. We modeled the GN for the reproductive and winter seasons and projected them onto each other (inter-prediction), and also onto transient migratory periods. To measure niche breadth and their overlap, minimum convex polygons (MCP) were calculated for the climatic space. The niches of each of the two seasons were tested for similarity using the PCA axes of climatic variables. The geographic areas with optimal, suboptimal and marginal conditions were identified, based on the distance to the centroid of the GN. The models for each season revealed no geographic inter-prediction among them, with the exception of winter to migratory seasons. The niche breadth of the winter was greater than that of the reproductive season, with an overlap of 22.47% and 45.18%, respectively. The similarity analyses showed a value of zero between seasons. The climate conditions for the records during the migratory months corresponded with suboptimal and marginal conditions of the sparrow’s winter niche. These results suggest that *A*. *bairdii* uses different climate conditions within ecological niches of each season during its migratory cycle.

## Introduction

The Grinnellian niche is defined as the multidimensional set of environmental conditions that allows a species to establish and persist [1–4]. Maguire [5] suggested that within the niche there is a structure created by variations in the combination of conditions that make it possible to identify distinct ecological regions; those closest to the niche centroid are considered optimal, since theoretically, correspond to higher values of the species breeding success, abundance and survival. The intermediate regions would be considered suboptimal and, while those located on the periphery of the niche are considered marginal. Unfortunately, in spite of the knowledge we have about the existence of the internal structure of niches and its effect on different aspects of species biology [6–10], the information on conditions used by migratory species during their non-breeding seasons is too limited.

Most migratory birds spend more than half of the year in non-breeding areas [11–15]; however, there is a marked bias in the studies on their biology, natural history, habitat selection, etc. toward the areas where these birds breed, at the northern extreme of their distribution [16–20]. This has resulted in gaps of information on migration and over-wintering population parameters for these species, which are essential since directly affect their annual survival and reproductive success [21–25].

It has been suggested that there are two basic patterns in the way migratory birds use climate conditions throughout their annual cycle. Some utilize the same conditions in breeding and wintering grounds (niche followers), while others change their use of conditions between seasons (niche switchers; [24,26–29]); although these definitions do not consider migratory transitional grounds. To understand the dynamics of use of the macro-climatic conditions of the reproductive and wintering areas, it is necessary to understand the relationship between the geographic areas used by a species throughout its annual migratory cycle and its environmental space, i.e. its Grinnellian niche [30–32]. Understanding this is essential to both, increase our understanding of migration and to aid in the definition and preservation of transitional areas and climates used only temporarily by these species [27,33–36].

Ecological niche modeling (ENM) is a technique that correlates the location records of a species with environmental conditions, making possible to reconstruct its Grinnellian niche, which is then projected onto the land. This enables the identification of sites with environmental conditions favorable for the potential presence of the species [37–39], even if records are scarce, since ENM is assumed to be accurate and reduce biases by fulfilling information gaps from non-sampled areas and periods [40]. ENM has also been used widely and with reliable results to analyze the niches of migratory bird species, for both their reproductive and wintering seasons [27,28,32,34,41–46].

Baird’s sparrow (*Ammodramus bairdii*) is a migratory species and a grassland specialist, whose population has been continuously decreasing over the last five decades [47,48]. It is assumed that such trend is associated with the loss of up to 80% of its reproductive and wintering habitat mostly due to agricultural activities [49–55]. It breeds in mixed-grasslands and fescue prairies of the northern U.S.A. and southern Canada, and winters in the grasslands of the Chihuahuan Desert in southern U.S.A. and northern Mexico. There is fine scale information about the natural history, ecology, and specifically habitat of *A*. *bairdii* for the breeding grounds [18,56–59]; however, studies addressing its winter ecology are scarce despite the fact it spends more than 50% of its annual cycle in these southern locations [12,60,61]. Knowledge on its migratory routes and habitats is even scarcer and limited to a reduced number of observational/curatorial records during this periods [62,63]. In addition, macro-climatic conditions have not been described throughout its annual cycle, and represent indispensable information to better understand and attenuate threats to this declining population due to climate change [22,47,64–66].

The objectives of the present study were to: 1) characterize the climate niches of the reproductive and winter seasons of *A*. *bairdii* through ENM; 2) analyze the use of these Grinnelian niches during the migratory stages, according to its internal structure (marginal, suboptimal and optimal conditions); 3) define and compare Grinnelian niche breadth for the reproductive and winter seasons; and 4) determine the climatic similarity between the two seasons.

Given that *A*. *bairdii* is a grasslands specialist and its environmental requirements during the reproductive and winter seasons are highly specific [18,56,60,61,67], we hypothesized that the climate conditions described for the different stages throughout its annual cycle will remain the same (niche follower, *sensu* Nakazawa [27].It was therefore predicted that the reproductive and wintering niches will show a high degree of overlap (greater than 50%), their projection of the optimal zones during migratory season will coincide with the species known distributional records, and climatic niches will not be dissimilar.

## Material and methods

### Presence and environmental data

We obtained monthly presence data for the species from electronic gazetteers, such as the Global Biodiversity Information Facility (GBIF); ORNIS, via VertNet (http://www.ornisnet.org/); unpublished databases curated by Bird Conservancy of the Rockies (BCR); Universidad Autónoma de Nuevo León (UANL); and the Integrated Monitoring in Bird Conservation Regions (IMBCR) Program. We only used records that were spatially and seasonally unique. We also verified localities and dates, based on the species known range, and eliminated any dubious (imprecise or unverifiable) records. As with most species [68], too few observations and specimens were available, and may be biased by site accessibility. Besides, Baird's sparrow, as many other species of conservation concern, has low occurrence information to cover satisfactorily their entire range; particularly during the migratory and winter seasons.

We compiled a total of 246 records for the reproductive (May to July) and 83 for the winter (December to February). We separated both, records and climate variables per month, since it has been proved its usefulness to describe the climatic conditions used by migratory birds due to the changing and transitional nature of the migration periods [31,34,69–71]. We gathered 28, 20, 33 and 15 records for April, September, October, and November, respectively. March and August were excluded from the analysis because are transitional months between seasons and record assignation to a particular period was not possible with confidence. These records; however, did help to delimit the reproductive, and migratory records.

To characterize the niches, three monthly climate cover layers (geospatial climate data) were used: maximum and minimum temperature and precipitation, with a resolution of 0.0416° (~5 km^2^), which were created by monthly interpolations of meteorological data from around the world from 1950 to 2000, and are known as WorldClim project [72]. Each layer is a raster (pixel or cell based file) with values of the corresponding climate variable assigned on each cell.

Of the 329 occurrence data points used for the model, 39 were obtained previous to 1950 and 217 from 2001 to 2013; thus only 73 records coincided with the climate data period. However, we used the whole set of data with confidence, considering that, global average increase in surface temperature from 1951 to 2010, was of approximately 0.6°C to 0.7°C [73], fluctuations in the species distribution are “short-term” (1890–2013), and that scenopoetic variables have slow dynamics, and can be considered static over many decades or more [38].

### Ecological niche models

We analyzed and generated two independent ENM for the reproductive and winter seasons [27,32,35,36,41,42,74–76], given that migratory bird can experienced different climatic conditions during their annual cycle [74,77,78] and most of them have distinct extent-of-occurrence (i.e. geographic distributions).

For the former, we used layers and records from May to June, since these months correspond with the optimal conditions for breeding with the highest vegetation productivity, food resources availability, and weather suitability [57,63,79–82]. For the second model we used records from December to February, when the species generally uses dense grasses, and depends on a enough seed availability that does not limit its winter survival [62,83–85], and suitable weather, with enough precipitation that optimizes the species' feather molt [86]. No ENM was generated for the migration months because of the low number of records and the uncertainty of the possible models.

To generate the ENM, we used the Genetic Algorithm for Rule-set Production (GARP), which is an evolutionary algorithm based on artificial intelligence that combines groups of rules for the reconstruction of ecological niches [87,88]. GARP uses known localities of species occurrence and environmental variables, to produce a model of the species geographic distribution, by relating the locality records of the species with environmental variables, in an iterative process of random rule selection, evaluation, testing and incorporation or rejection [89]. This algorithm has been found to be more effective during transfer to different scenarios [90,91]. Transferability refers to apply a model developed on one scenario to another scenario or to another time in the same area [38].

Algorithm performance was optimized running 100 replicates, selecting the ten best models based on the lowest rates of omission error (proportion of pixel raster of know presence of the species that are predicted absent by the model; maximum 10%) and intermediate rates of commission error (measured as the median of the pixels that are predicted as presence by the models; this is because we have no true absences of the species) [92]. For both seasons, 80% of the records were used to calibrate the models and 20% to evaluate them; this selection was made randomly and out of the program. The evaluation of the model performance was developed using a variant of ROC curve called partial ROC [93] using the Tool for Partial-ROC V.1.0 [94]. The partial ROC analysis generates proportions or ratios with values that range from zero to two, from the proportions described through the ratios of correct identification of presences against the total area predicted by the algorithm [93]. The ratios with values close to one describe a behavior that is similar to chance, and those closer to two, suggest a perform better than random. The partial ROC utilizes the partial area of distribution, which provides a stronger basis for evaluation of the ecological niche model predictions [93], giving more weight to omission errors than to commission errors; and providing predictions with acceptable levels of the former [93]. In contrast, the traditional ROC curve has been criticized [93,95], since underestimates the models that do not provide predictions across the spectrum of areas proportional to that of the study area, and also produces an inappropriate standardization of the weight of the errors of omission and commission, which is dependent on the total area used for modeling.

The distance from each pixel to the centroid of each niche was used as a measure of the internal structure of the niches (reproductive and winter seasons). Based on Maguire’s [5] description, niche structure fitness improves as distance to the centroid decreases; thus, there is an array of environmental regions within each niche that are characterized by optimal, suboptimal and marginal climate. In the present study, in contrast to Maguire’s [5] proposal, no measures of fitness were taken because it would represent a very expensive and time consuming effort; thus, we only described the internal structure of the niches with respect to their distances to the centroid, though to do so the description of each region as optimal, suboptimal or marginal was retained, in order to have reference points for the different environmental areas within the structure of the niches for the reproductive and winter seasons. To this end, the ecological distance of each pixel to the niche centroid was calculated for each season, using climate variable values corresponding to the cells predicted as potential presence (modeled niche). In order to unify units, each variable was Z standardized (mean = 0, standard deviation = 1), subtracting the mean from each value of the variable and dividing it by its standard deviation, where the niche centroid was the value of the variables that were equal to zero. Once the niche centroid was estimated, the multidimensional Euclidian distance from each pixel to the centroid was calculated, using the formula:

$$DC=\sqrt{\sum_{i=j}^{n}{\left(y_{ij}-{\bar{y}}_{j}\right)}^{2}}$$

where DC is distance to the niche centroid, y_ij_ is the value of variable *j* in population *i*, and ӯ_*j*_ is the mean of variable *j* [6,7]. The result was a raster with distance values that was reclassified into the categories of optimal, suboptimal and marginal based on more parsimonious cut-offs: optimal (0.043–1.51), suboptimal (1.52–2.9), and marginal (3–4.4). This process was carried out in ArcView 3.2 [96].

### Niche breadth

We used the range of climate conditions described for the geography, based on the presence of individuals of the species (effective niche; *sensu* Soberón [4], Quintero and Wiens [97]) to determine niche breadth for each season and migratory transient months, following a two-step process. The first was to estimate the polar coordinates based on a climate profile of the areas predicted by the models. The polar coordinates consider the different variables as force vector, so that each polar coordinate, X and Y of each season, would be the equilibrium point of all vectors, in other words, of all variables. The component of each vector would be the value of the variable and the angle of each vector would be an assigned value to each variable. Hence, X and Y coordinates, representing all variables used to define the niche of each season, can be plotted [98]. This was done using the EnvNicheR library [99] from R [100].

The second step was to calculate the Minimum Convex Polygon (MCP) described by the polar coordinates for each stage of the cycle, following the method proposed by Pateiro-López y Rodríguez-Casal [101]. This technique identifies the most convex polygon that avoids overestimating the occupied climate area [102]. The area of each MCP was calculated using the *alphahull* package [103] in R [100], which was considered as niche breadth, such that larger areas were interpreted as wider niches.

### Comparison and similarity of seasonal niches

In order to define the similarity among the seasonal niches of the species, we compare their niches through seasonal inter-projection, overlap, and a similarity test as follows.

#### Projections

Models generated for each season were projected onto each other conditions to test their inter-prediction power and consequently their climatic equivalence. Projections were also made onto migratory months (September, October, November and April).

#### Overlap

Overlap of niche breadth, as previously defined, was measured as a percentage of pixels coinciding for both MCP polygons integrating climatic niche, this was done using the EnvNicheR library [99] from R [100].

#### Similarity analysis

To analyze whether there were any differences between the winter and reproductive season niches [104,105], we used the niche similarity test proposed by Broennimann et al*. [104]*, running the Ecospat package [105] in R [100] and using the PCA-env (PCA calculated on the climatic space). The principal components were calibrated with modeled conditions from both seasons (breeding and winter) which were then associated with occurrence densities on each season. Once the climatic space area is described by the first two PCA axes, the estimated relative species occurrence densities (from a kernel density function) and the relative frequency of the environmental conditions from each season are mapped onto it. For the similarity test, the overlap between the niches was measured using an observed value of Schoener’s *D* index [106,107] on the occurrence densities, which ranges from 0 (complete differentiation) to 1 (complete similarity). To contrast and interpret the observed values of *D*, the test also generates null models by recalculating the *D* overlap values for randomly selected records from the records available for both seasons 100 times; with this information a histogram of estimated (null) values is plotted to compare them with the observed values.

#### Migratory route comparison

In order to determine the conditions of the niche used by the birds during their migration, climate profiles were created for the existent monthly records during this period. These profiles were compared with those from each modeled season and from the projections onto the migration months.

## Results

**Fig 1:**
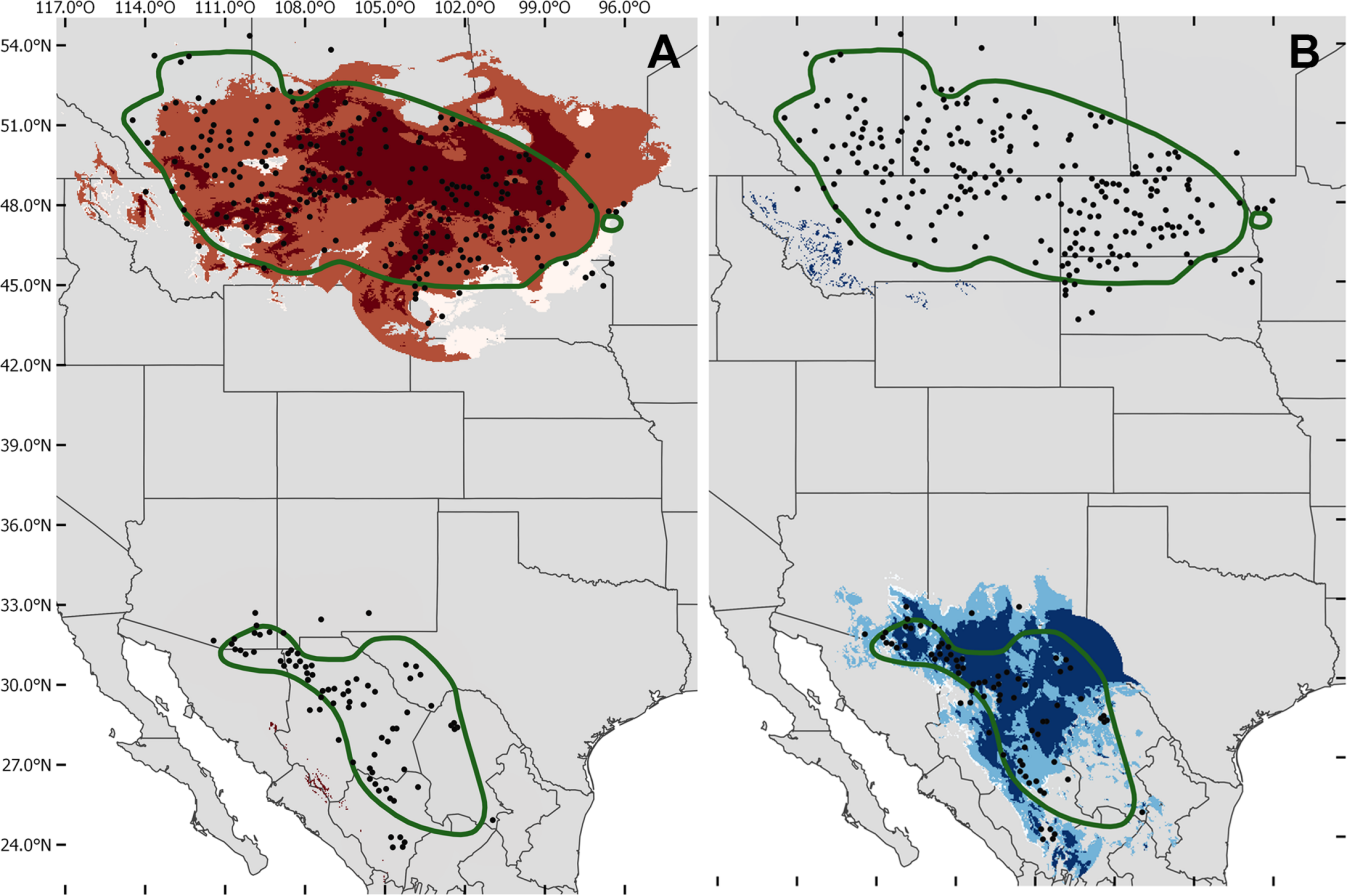
**Area predicted for the reproductive season and its projection onto the winter zone (A) and area predicted for the winter season and its projection onto the reproductive zone (B)**. The green polygons represent the known distribution of Baird’s sparrow, according to the IUCN. The northernmost polygon is the reproductive range and the southernmost is the wintering one. Darker colors indicate optimal niche conditions based on their proximity to the centroid.

**Fig 2:**
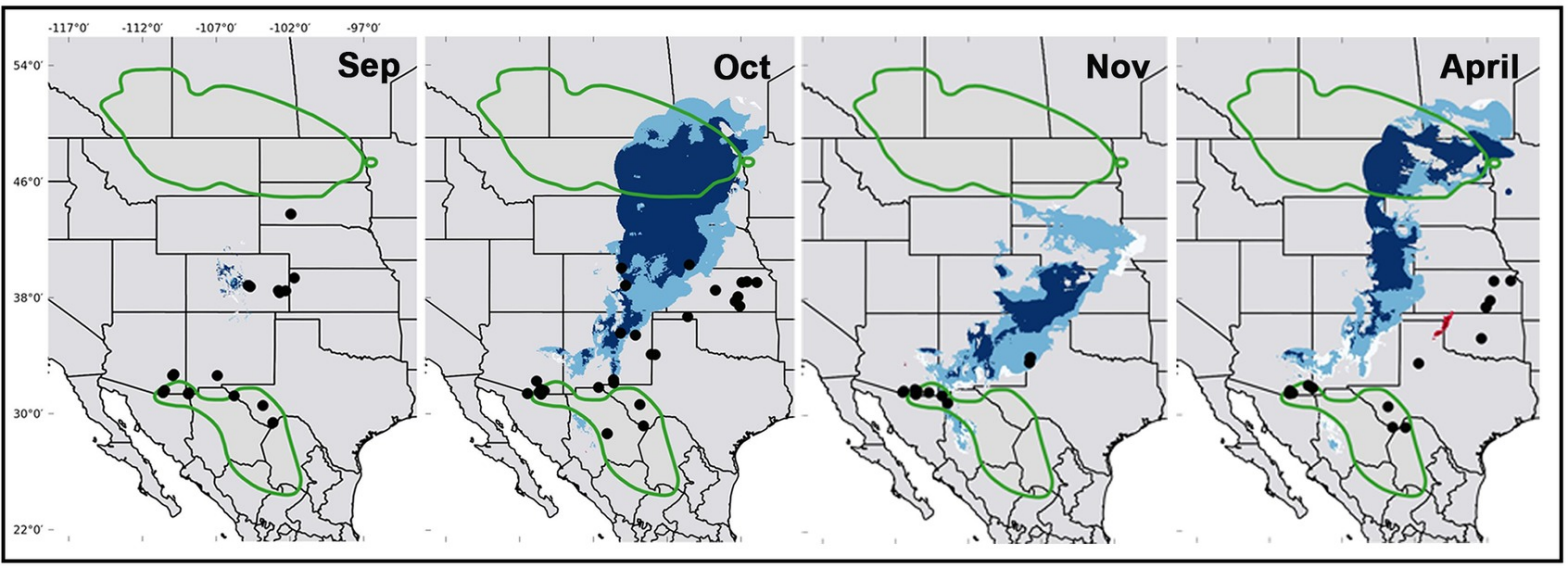
Area predicted by the projection of the conditions of the reproductive season (red) and winter (blue), onto the months of the migratory route of Baird’s sparrow. For the month of September the reproductive season conditions were not projected. Darker colors indicate optimal niche conditions based on their proximity to the centroid. Dots correspond to the record localities for each month.

The generated models, according to the evaluation with the partial ROC, were statistically better than random for both seasons, reproductive (AUC ratio = 1.26; p = 0.001) and wintering (AUC ratio = 1.30; p = 0.001). There were no overlapping areas in the inter-predictions made for both seasons (Fig 1A and 1B). However, they did showed predictive power for migratory months areas. Winter model predicted larger geographic areas of at least 0.6% on the migratory transient zones, while the reproductive model predicted a maximum of 0.3% (Table 1, Fig 2. S1 Fig). It is also worth noticing that winter conditions showed a pattern of movement from north to south as months approach winter season (Fig 2, S1 Fig).

**Table 1:** Percentage of overlap between the minimum convex polygons (PMC) of the bioclimatic conditions models by season and projections to the migration months of *Ammodramus bairdii*.

Seasons	Overlap seasons-migration months (%)	Migration Months	Overlap migration months-seasons (%)
Winter	18.14	September	83.5
Winter	58.11	October	55.13
Winter	59.81	November	59.81
Winter	83.75	April	70.33
Reproductive	NA	September	NA
Reproductive	0.24	October	25
Reproductive	0	November	0
Reproductive	1.03	April	10.81

**Fig 3:**
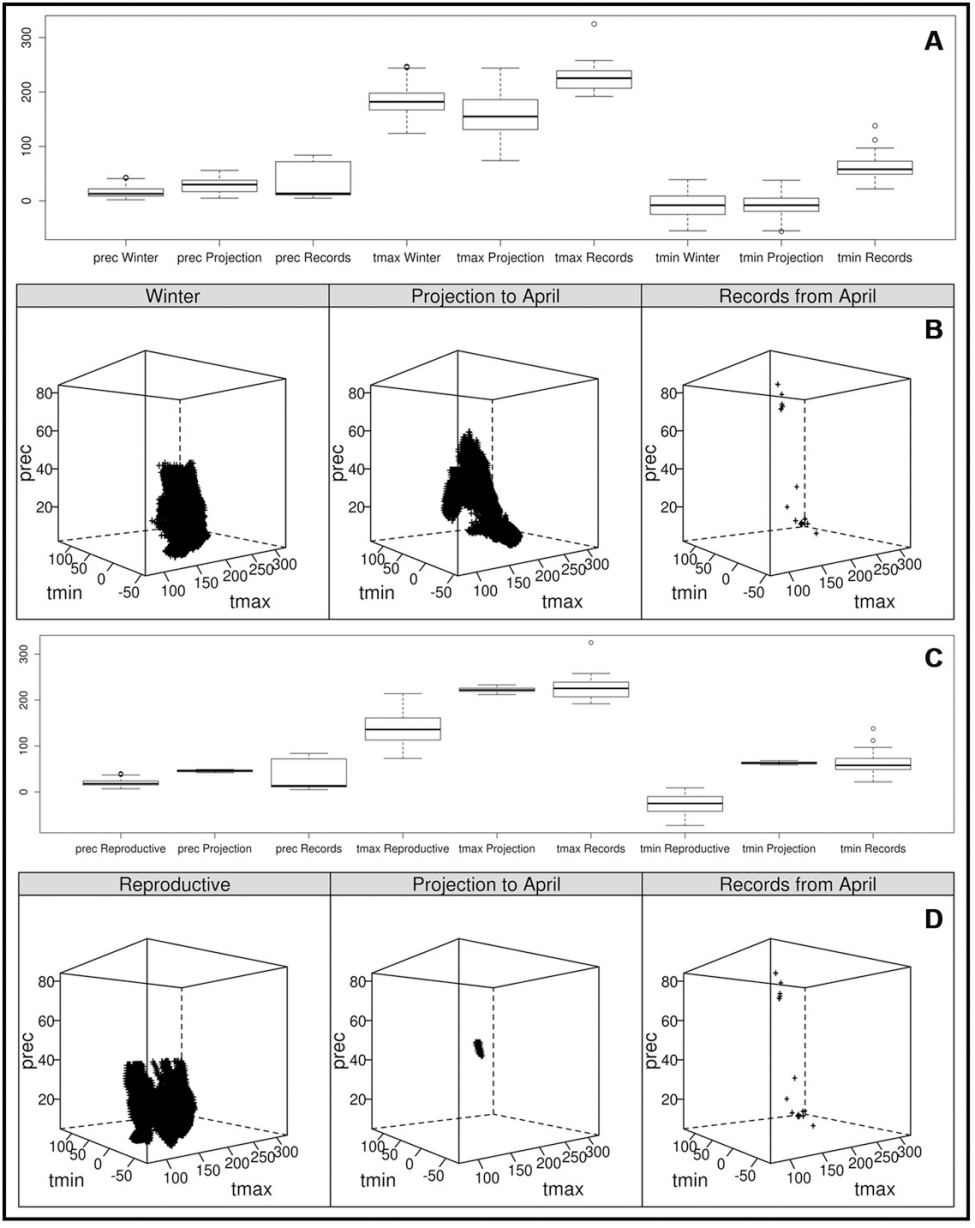
**Comparison of the climate profiles of the winter model (panels A and B) and the reproductive season model (panels C and D) for Baird’s sparrow, record localities, and projection onto the month of April (prec = precipitation, tmax = maximum temperature, tmin = minimum temperature)**.

The climate profiles during and throughout the migration route, described by the locality records for each transition month, had little in common with the areas predicted by the projections for winter; October was the month with the most records predicted (22%), and even less with those for the reproductive season, where no record was predicted. In the cases for which the locality records coincided with the projections–which were mainly those of the winter–the record sites were located in suboptimal and marginal zones of the niche (Fig 2). Comparisons among the climate profiles (for the presence records for each month, for the projections of the reproductive season and winter onto the migratory passage months, and for the models for each season) revealed that the variable that differs the most is precipitation, with the record sites having the highest values except for the month of November (Fig 3; S2 Fig and S3 Fig). Thus, records of *A*. *bairdii* were denser in climates with more precipitation in the winter and in climates with warmer temperatures during the reproductive season (Fig 3) that in both cases might be correlated with vegetation cover and ultimately favour the species density.

**Fig 4:**
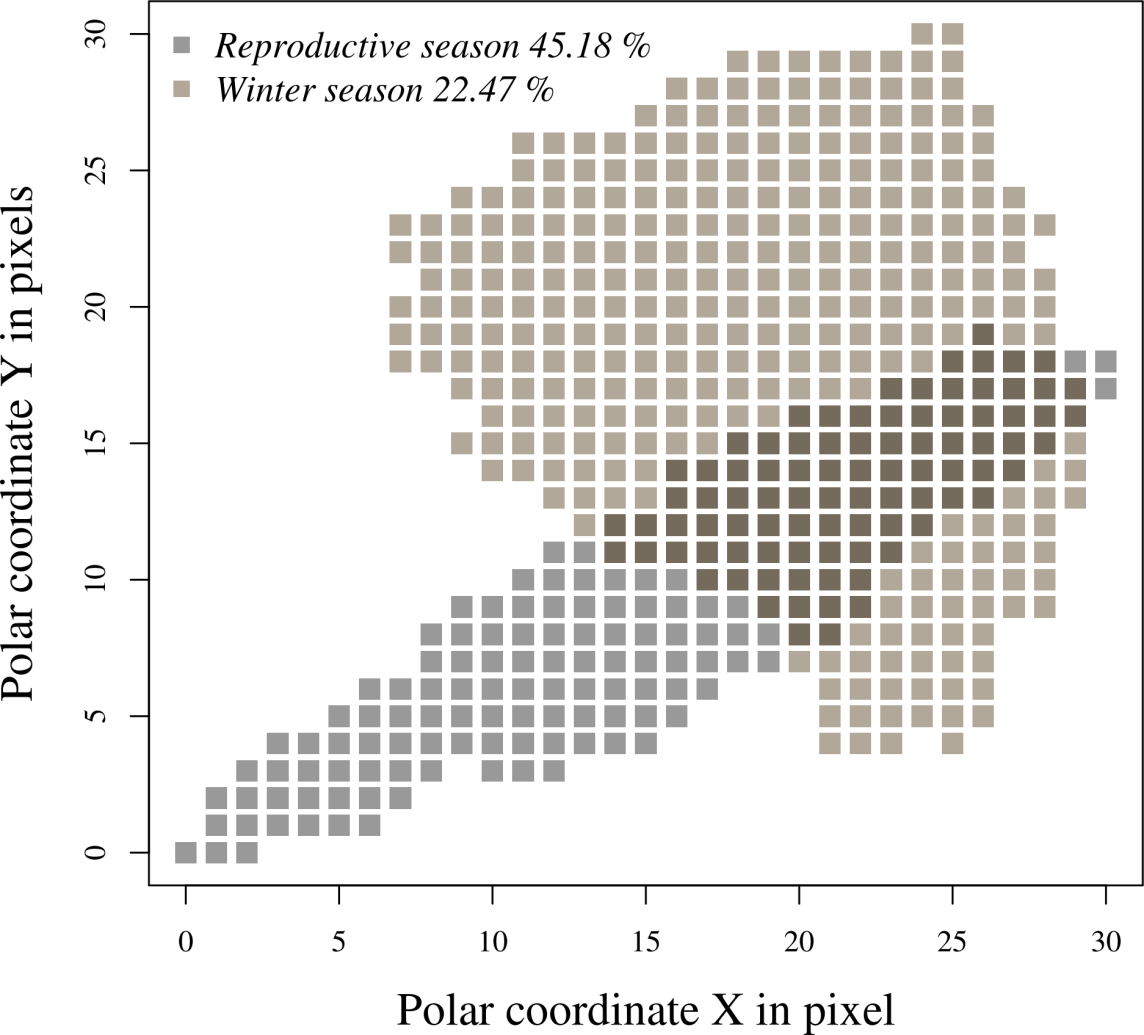
Percent overlap of the minimum convex polygons (MCP) for the polar coordinates of the reproductive season and winter of Baird’s sparrow. Polar coordinates were created based on the climate profile for each model.

Niche breadth was larger for the winter season (346.6 pixels) than the reproductive one (165.3 pixels). There was an overlap of 45.18% of the climatic niche of the reproductive season with the wintering one, and of 22.47% vice versa (Fig 4).

**Fig 5:**
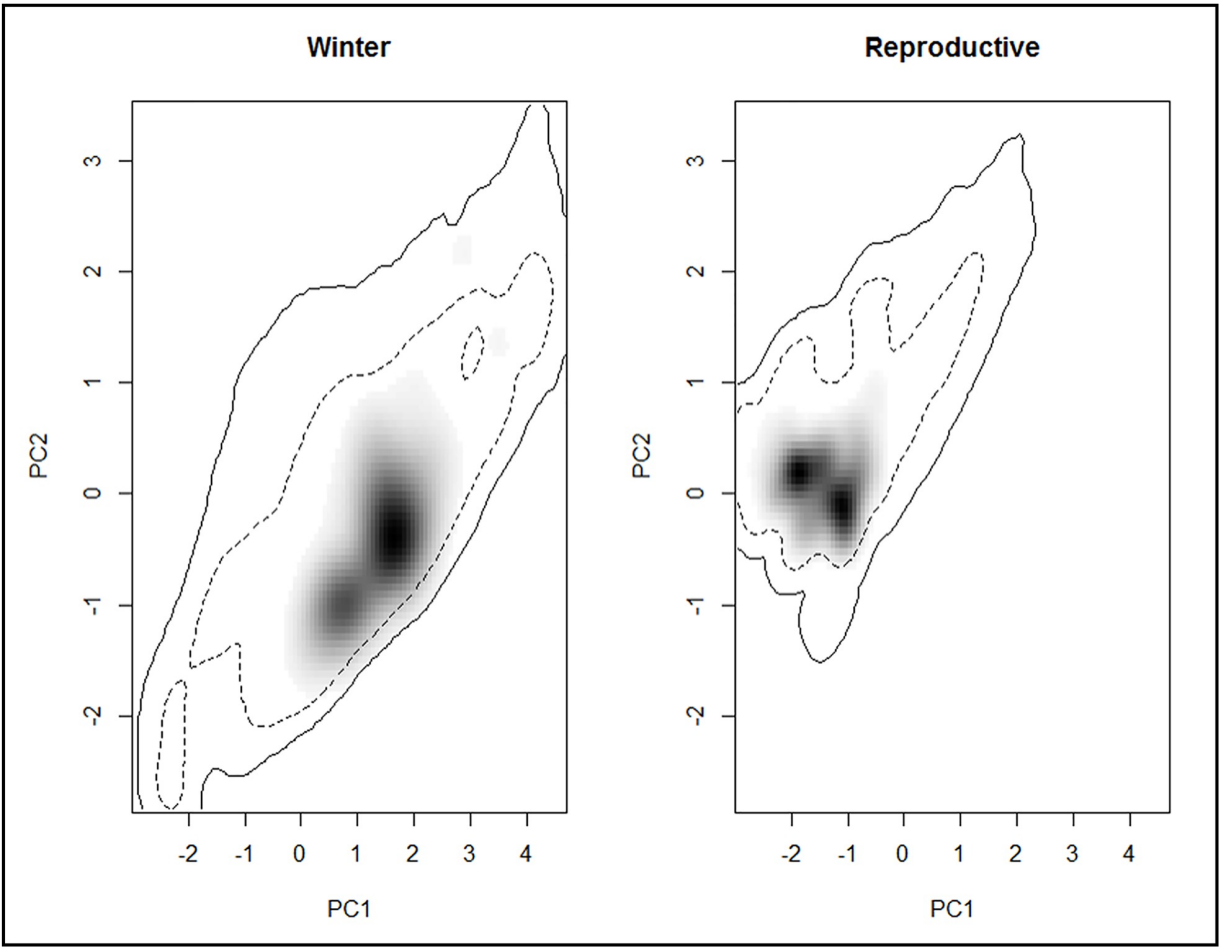
Conditions of each seasons' niche occupied by Baird’s sparrow. The niche is represented by the first two PCA axes. Gray shading represents the density of occurrence of individual birds in the climatic space. The dotted line and the solid line represent 50% and 100%, respectively, of all of the environment available to the species.

**Fig 6:**
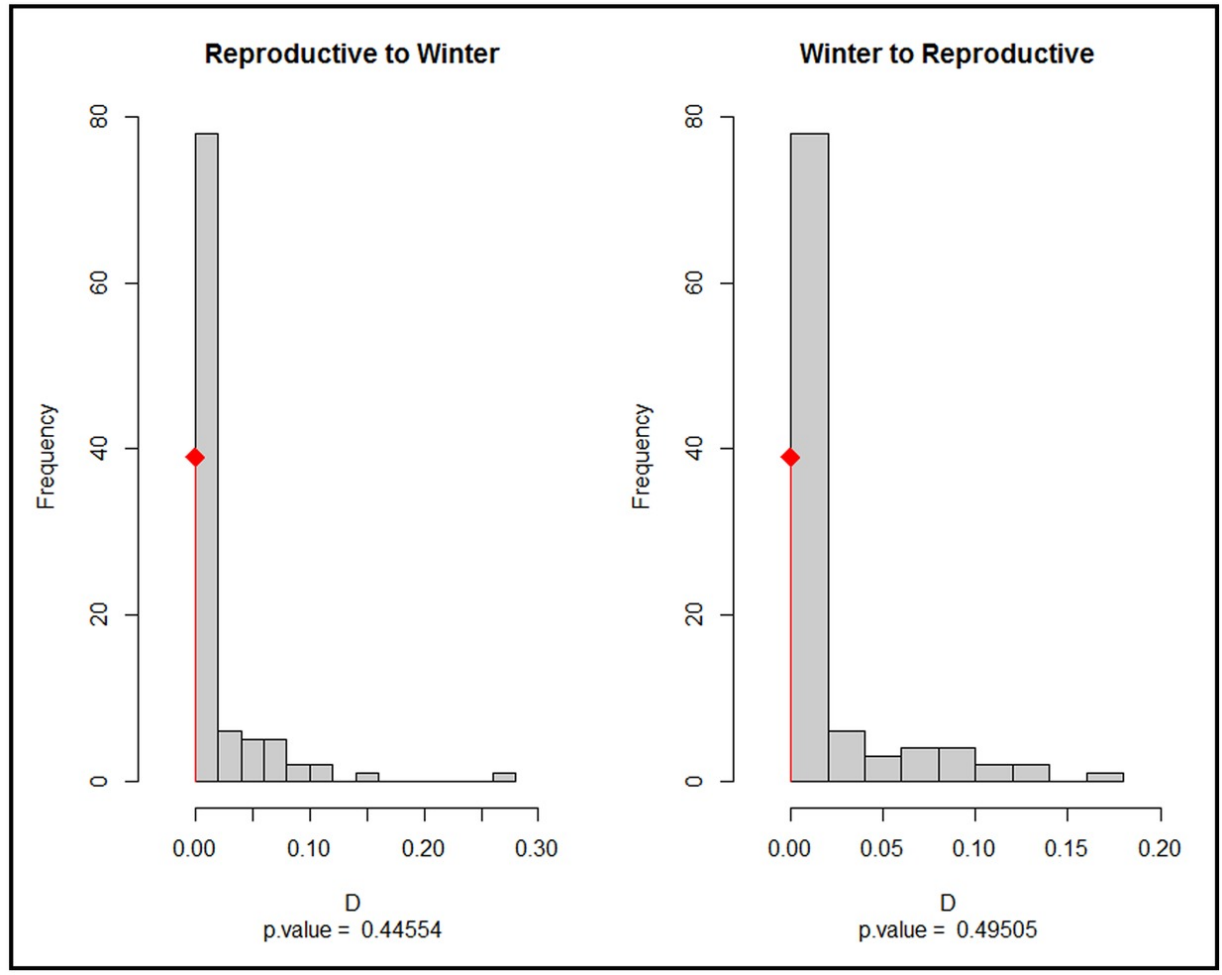
Histograms of observed overlap for the *D* index (Broennimann et al. 2012) between winter and the reproductive season (red lines) data for Baird’s sparrow with the null model (gray bars) for the niche similarity test. The histogram corresponding to the niche similarity of the winter niche in the reproductive zone (right) and the similarity of the reproductive season niche in the wintering zone (left).

Niches (winter and reproductive) were not more dissimilar than expected by chance for either the reproductive season niche onto the winter niche, or the winter niche onto the reproductive niche (*D* = 0; p = 0.44 and p = 0.49, respectively; Figs 5 and 6).

## Discussion

It is currently irrefutable that climate exerts a huge effect on the spatial and temporal distribution of species, especially in sites where there are marked seasonal changes throughout the year [108,109]. It has even been suggested that this variability in climate is one of the main reasons, whether direct or indirect, for birds migration, allowing them to live in different climate conditions during the reproductive and winter seasons, according to their physiological needs on each one [27,36,110–112].

Our results show that the patterns described for *Ammodramus bairdii* are complex in terms of climate. For example, 77% of winter niche breadths for the reproductive and winter seasons did not overlap, suggesting that *A*. *bairdii* is a niche switcher (*sensu* Nakazawa et al. [27]); however, their dissimilarity did not perform better than chance (*D* = 0 in both directions). Also, the projection of the winter model toward the transient months revealed a wider climate (and geographical represented) niche. There is an accepted idea that temperate species have wider niches [113] in response to strong climatic seasonality [97]. In contrast, the transfers of the reproductive conditions toward the transition months indicated greater spatial restriction.

Recalling that the niche of the species is the sum of conditions used throughout the annual cycle, it is likely that the niche is greater than the one defined using only breeding and wintering seasons. Migratory transient months showed in all cases exclusive climate conditions, with no overlapping with the optimal conditions of the reproductive nor wintering seasons. In other words, the climate space (Grinnellian niche) used by *A*. *bairdii* is composed of partially differentiated climatic conditions during each stage of the migratory annual cycle. However due to the climate seasonality, the species only has access to the conditions present in the geographic space [114–116], hence it uses different climatic conditions in each stages (niche switcher).

Based on the structure of the climate niche, the migratory records were mostly associated with predicted marginal conditions. This could result from a directed migration strategy, probably due to characteristics other than climate, such as the wind, which has a huge influence on energy expenditure [117–119], or fine-scale factors (the hypothesis of Eltonian noise[120]), such as areas with vegetation characteristics that offer more feeding resources [56,60–62]] and allows avoidance of potential antagonistic interactions and predation [121–124].

Our results suggest that this species occupies different climate conditions during its annual cycle, although the migratory route has several knowledge gaps. Migratory routes analysis has received little attention in spite of its importance on the individual survival, molt, timing of arrival, reproductive success, population size and dynamics [13,86,125–128]. In fact, the specific geographic migration routes are not known for the majority of species, with only rough descriptions of their movements available. Furthermore, in spite of the role that climate could be playing in the process of determining migratory patterns, there are still too few studies describing and analyzing the climate niches for migratory routes [24,31], though this areas are critical for the involved populations, given the high mortality rates that occur along them [129].

Owing to the limited number of records for the transitional months, it is possible that the climate conditions used by this species over the course of its migratory cycle are not fully represented. But again, it highlights the importance of focusing efforts on studying the migratory routes of birds, especially when their habitats are particularly exposed to high rates of transformation and loss, as with the North American grasslands [49–55].

Now, despite there are different specific climate conditions on each of the stages of its migratory annual cycle, *A*. *bairdii* does maintain its use of grasslands throughout it [56,58,60–63], which is related with its selection of a specific type of vegetation, a component of its niche we did not include in our analyses, but that has been associated with nesting site selection, food availability, and predation risk, among others, and that ultimately affects its reproductive success [18,59] and overwintering survival [41,130,131]. Although the loss of 80% of grasslands in the Great Plains of North America [53–55] has played a major role on its population decline over the past five decades [48,66], probably climate change will increase the pressure on this ecosystem and its associated bird species on different aspects. Vegetation cover will be differently affected on the northern (declined in late summer) and southern (increased in autumn and winter) areas [132].

Changes in the precipitation variability will increase stress in plants and change towards a plant community with more xeric-affinity species [133]. Migratory birds are particularly prone to high risk under climate change [134–136], since it will alter migration timing: has been observed that short-distance migrants will arrive earlier in spring and later in autumn, while long-distance migrants will leave earlier on the autumn. For example, it has been report that more than a half of migratory species breeding at the northern Great Plains of North America arrived earlier at their reproductive areas in the last 10 years [137,138].

Climate change also will have an effect on the extent of habitat for birds, some species showing potential losses and other potential gains [132,135]. In the case of *A*. *bairdii* Peterson [65] have suggested a contraction of its breeding range, to current south-central area. Its winter distribution will also undergo severe changes, with local extinctions predicted for the Chihuahan desert [139], where a decrease of 6–11% of winter moisture is predicted in Mexico [136]. However, these analyses did not consider the seasonality of the climatic conditions, which are key to understand its annual cycle [42,45,69,71,75,78]. Thus is important to analyze the effect of climatic change on each stage of the annual migration cycle of the migratory grassland birds.

It is necessary to increase the knowledge about the biological processes involved, in order to understand species migration, including intra- and inter-specific interactions that intervene differentially depending on season, although mainly in overwintering and transitional areas. There is a lack of information not just for *A*. *bairdii*, but for most of the migratory grassland birds, with respect to its overwintering and migration areas about, among others, vegetation structure and composition, diet, survival rates and the factors affecting these [21,140,141]. Analyzing the relationship between the structure of ecological niches (*sensu* Maguire [5]) and the way they are occupied along the migratory routes, is fundamental to improve our understanding of the ecological patterns and processes that define the geographic distribution areas, and to understand the nature of the spatio-temporal relationship of the environment on different migratory species.

## Supporting information

S1 Fig Percentage of overlap of the minimum convex polygons (MCP) for the polar coordinates of each season and theirs projection to each transition month. The polygons were create from the climate profile of models and projection respectively.
(PDF)

S2 Fig Comparison of the climate profile of each season, their projections to each transition month and records from each month. (pre = precipitation, tmax = max temperature, tmin = min temperature).
(PDF)

S3 Fig Comparison of the climate profiles of both season model, projection onto the month and record localities from each month. (prec = precipitation, tmax = maximum temperature, tmin = minimum temperature).
(PDF)
