# Supplementary material for: Climate complexity in the migratory cycle of Ammodramus bairdii
Source: PLoS One. 2018 Aug 27;13(8):e0202678. doi: 10.1371/journal.pone.0202678 (PMC6110464; doi:10.1371/journal.pone.0202678)
Supplement: S1 Fig — The polygons were create from the climate profile of models and projection respectively. (PDF) [file pone.0202678.s001.pdf]

S1 Appendix. Percentage of overlap of the minimum convex polygons (MCP) for the polar coordinates of each season and theirs projection to each transition month. The polygons were create from the climate profile of models and projection respectively.

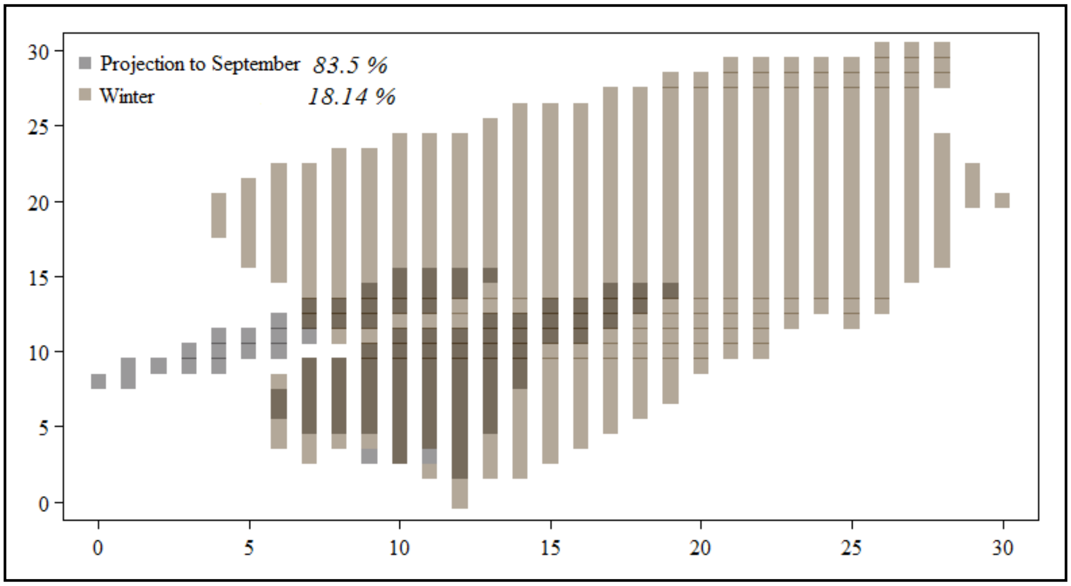

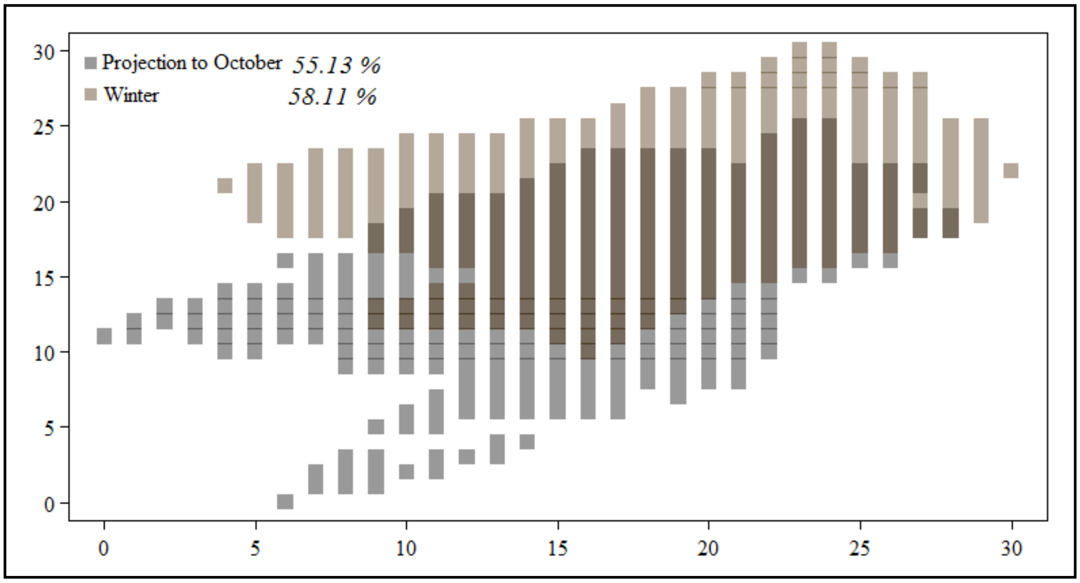

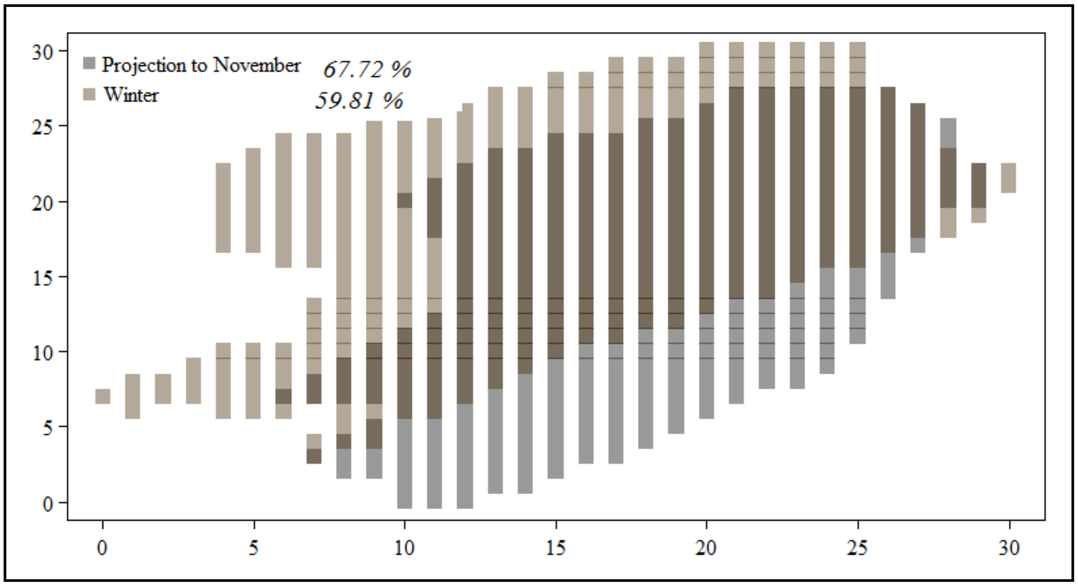
Projection to November 67.72 %
Winter 59.81 %
0
5
10
15
20
25
30

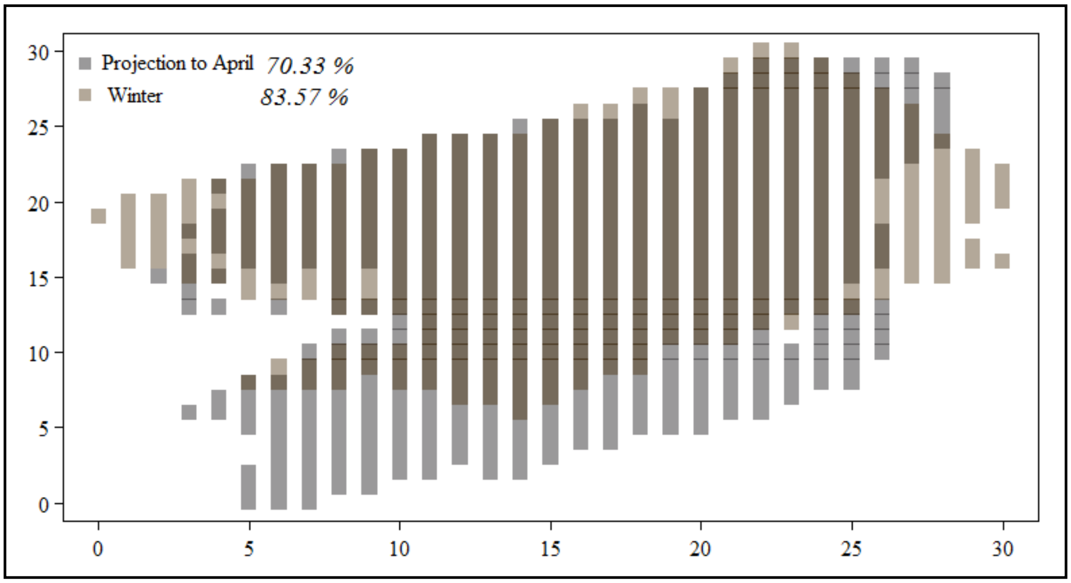
Projection to April 70.33 %
Winter 83.57 %
0
5
10
15
20
25
30

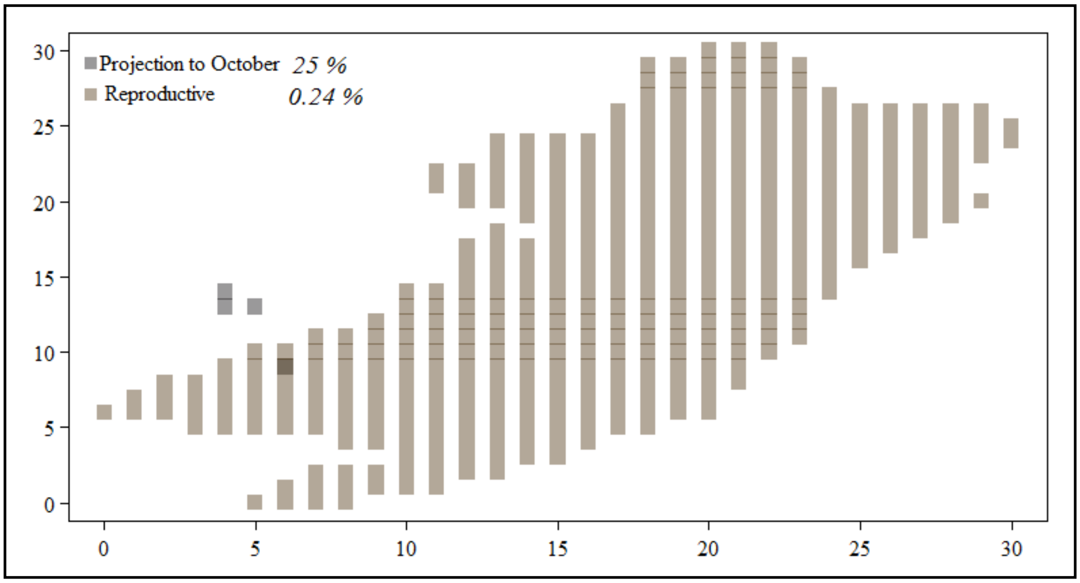
Projection to October 25 %
Reproductive 0.24 %
30
25
20
15
10
5
0
0
5
10
15
20
25
30

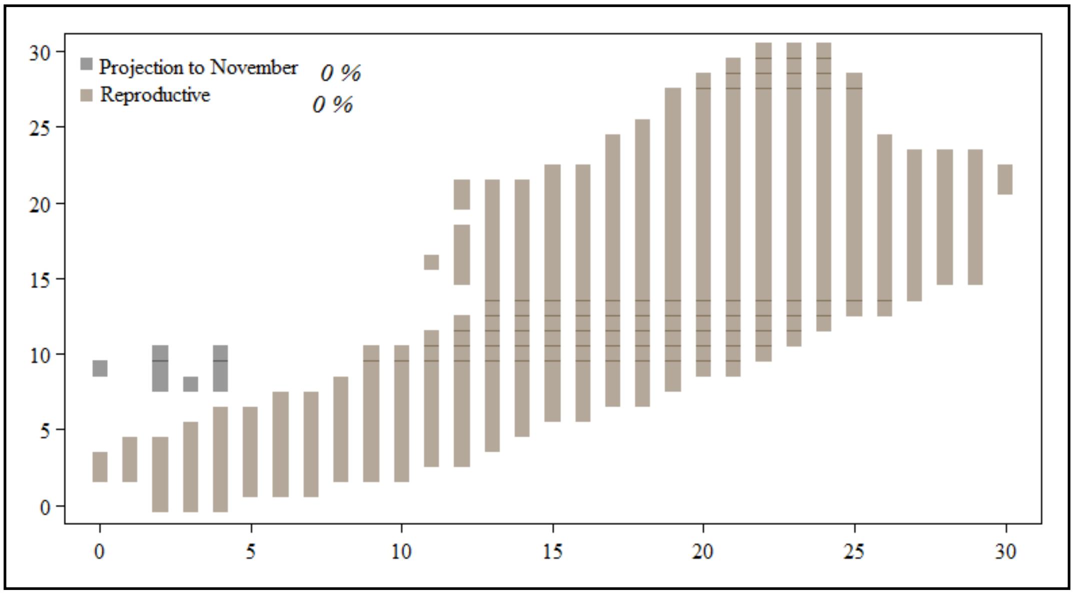
Projection to November 0 %
Reproductive 0 %
30
25
20
15
10
5
0
0
5
10
15
20
25
30

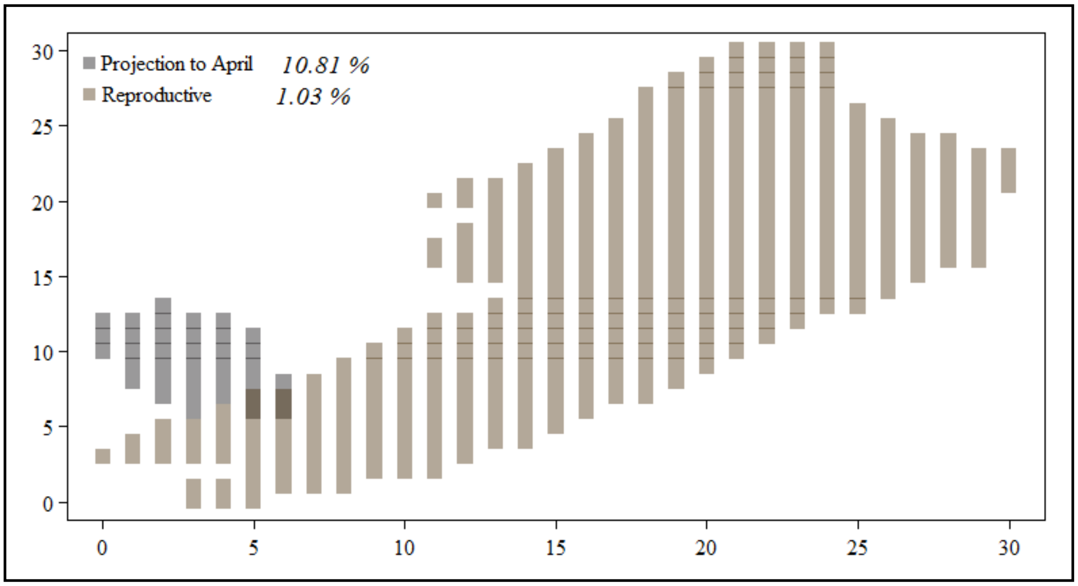
Projection to April
10.81 %
Reproductive
1.03 %
30
25
20
15
10
5
0
0
5
10
15
20
25
30
