# Supplementary material for: Climate complexity in the migratory cycle of Ammodramus bairdii
Source: PLoS One. 2018 Aug 27;13(8):e0202678. doi: 10.1371/journal.pone.0202678 (PMC6110464; doi:10.1371/journal.pone.0202678)
Supplement: S2 Fig — (pre = precipitation, tmax = max temperature, tmin = min temperature). (PDF) [file pone.0202678.s002.pdf]

S2 Appendix. Comparison of the climate profile of each season, their projections to each transition month and records from each month. (pre=precipitation, tmax= max temperature, tmin=min temperature).

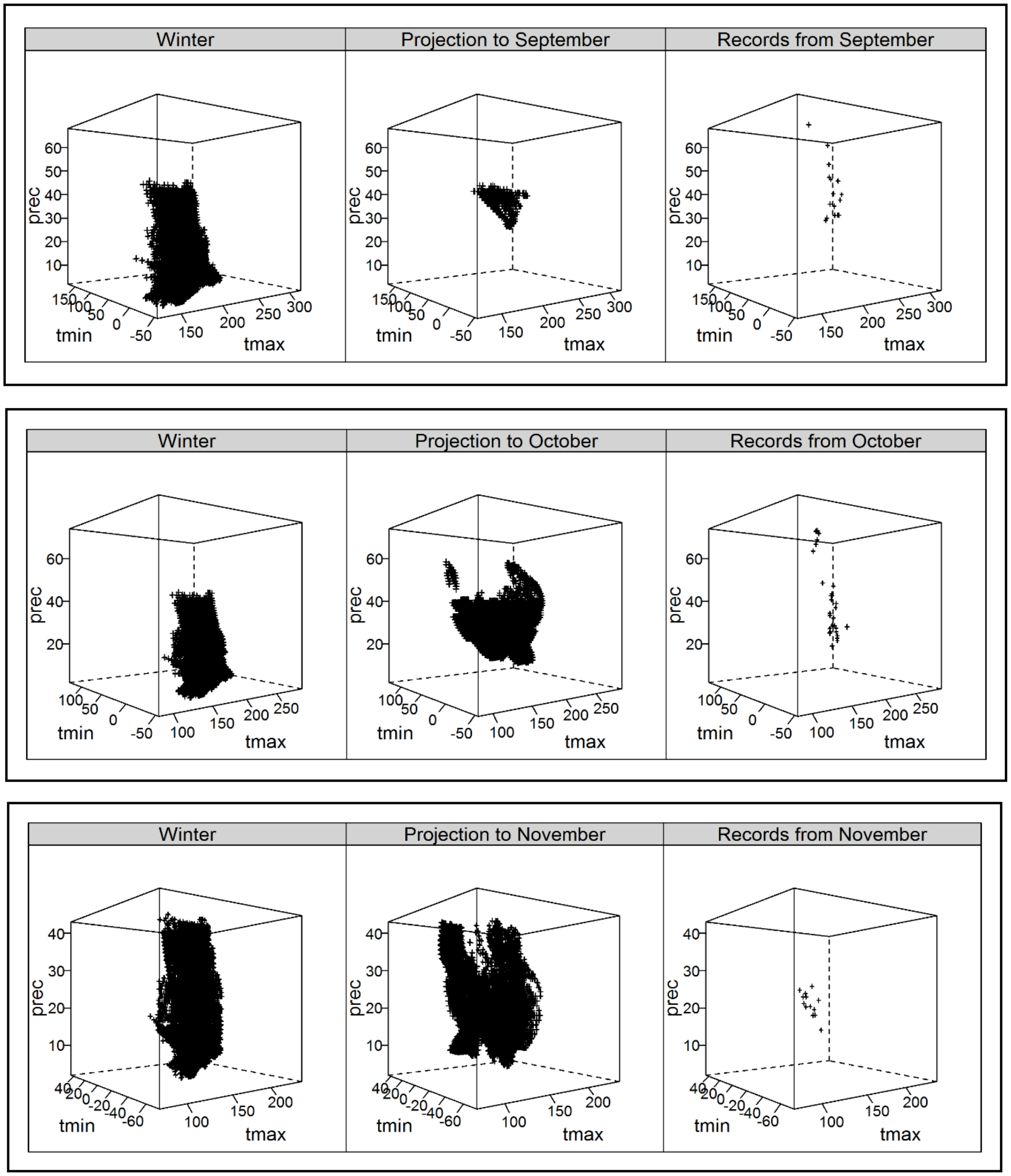

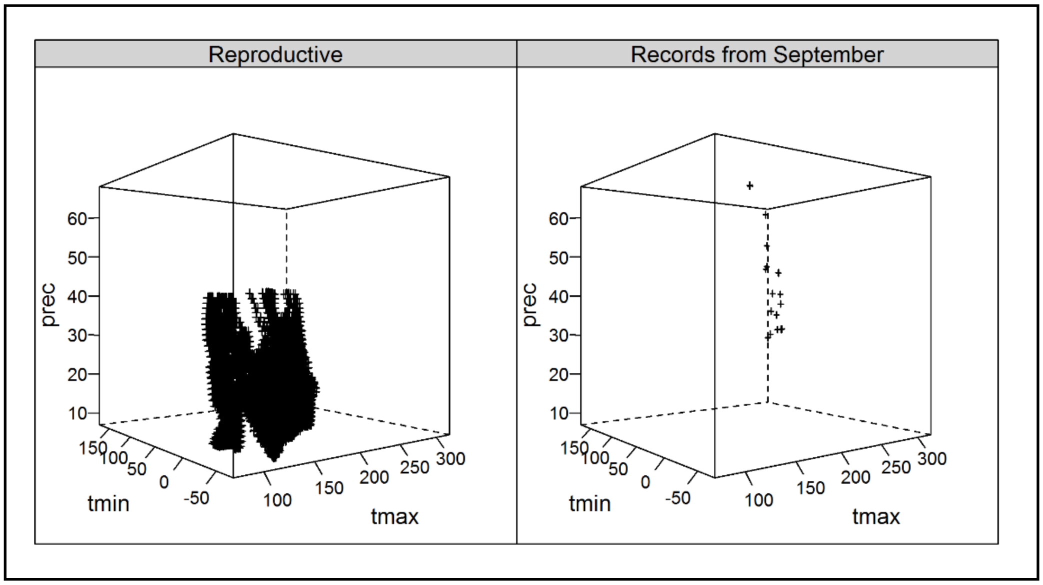
Reproductive
Records from September
prec
tmin
tmax

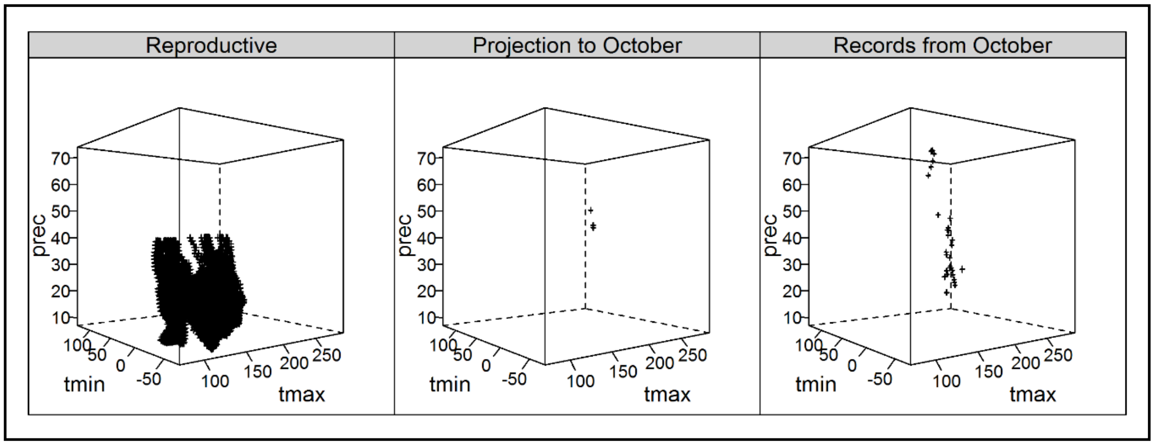
Reproductive
Projection to October
Records from October
prec
tmin
tmax

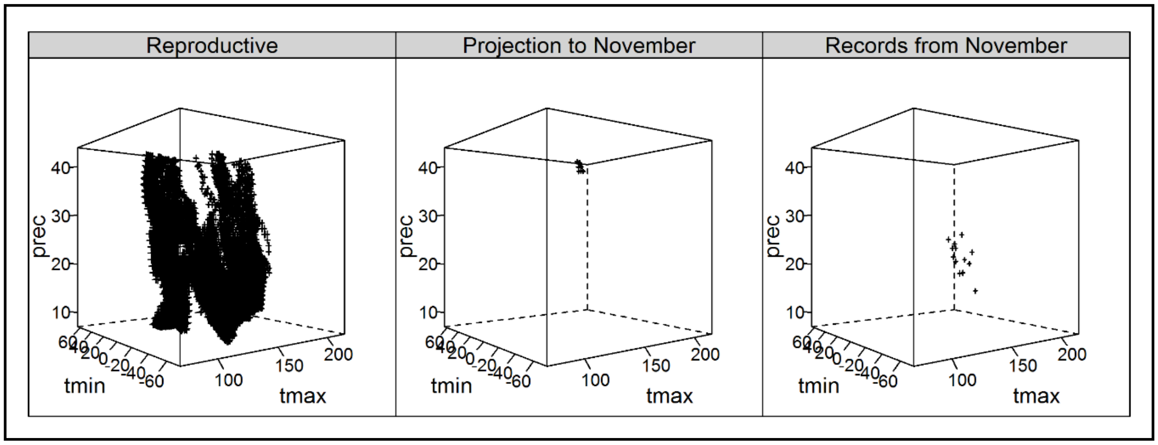
Reproductive
Projection to November
Records from November
prec
tmin
tmax
