# Supplementary material for: Climate complexity in the migratory cycle of Ammodramus bairdii
Source: PLoS One. 2018 Aug 27;13(8):e0202678. doi: 10.1371/journal.pone.0202678 (PMC6110464; doi:10.1371/journal.pone.0202678)
Supplement: S3 Fig — (prec = precipitation, tmax = maximum temperature, tmin = minimum temperature). (PDF) [file pone.0202678.s003.pdf]

S3 Appendix. Comparison of the climate profiles of both season model, projection onto the month and record localities from each month. (prec= precipitation, tmax= maximum temperature, tmin= minimum temperature).

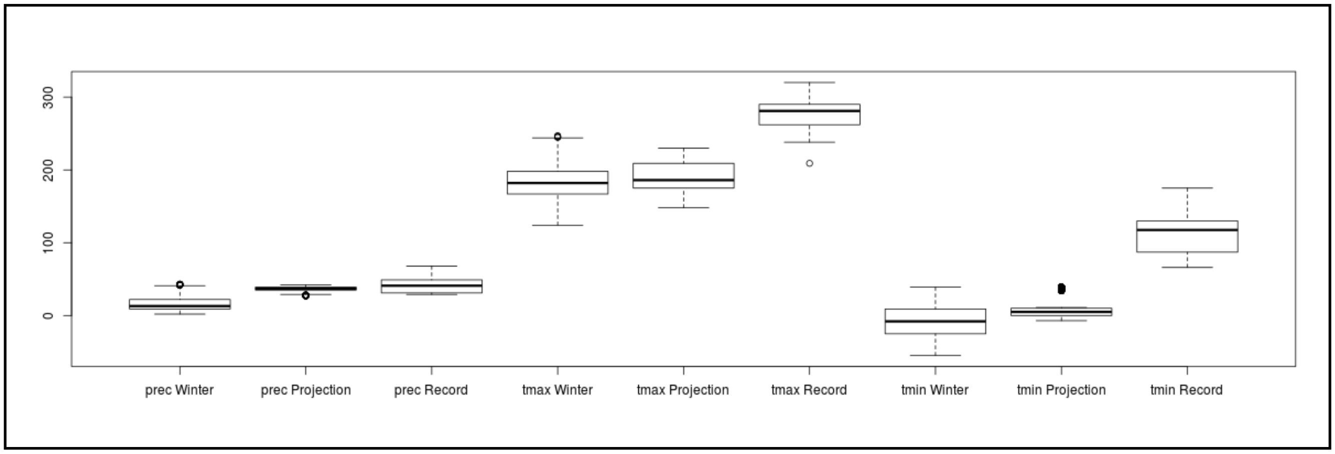

Winter to September

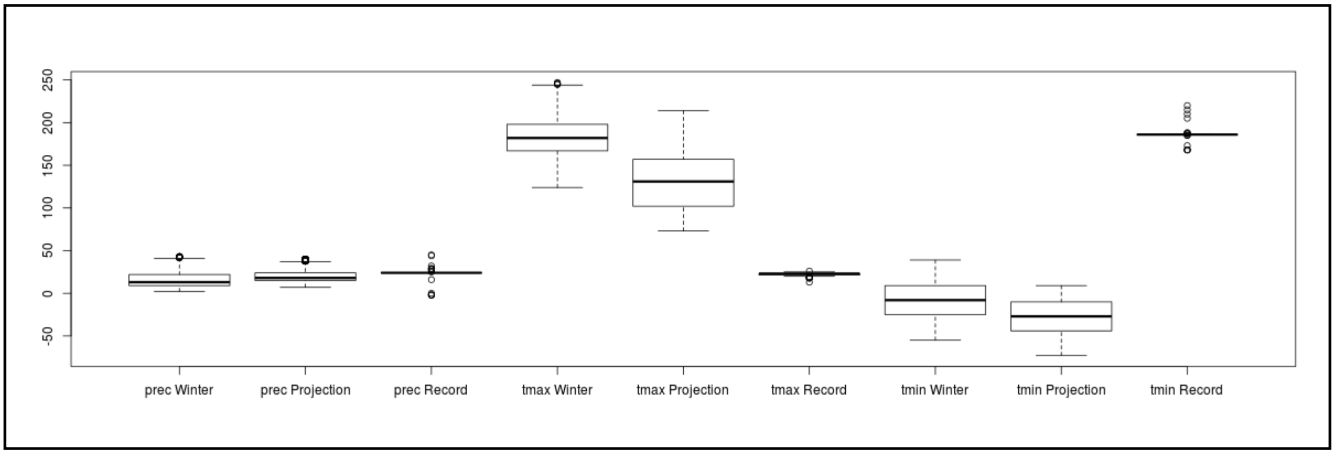

Winter to November

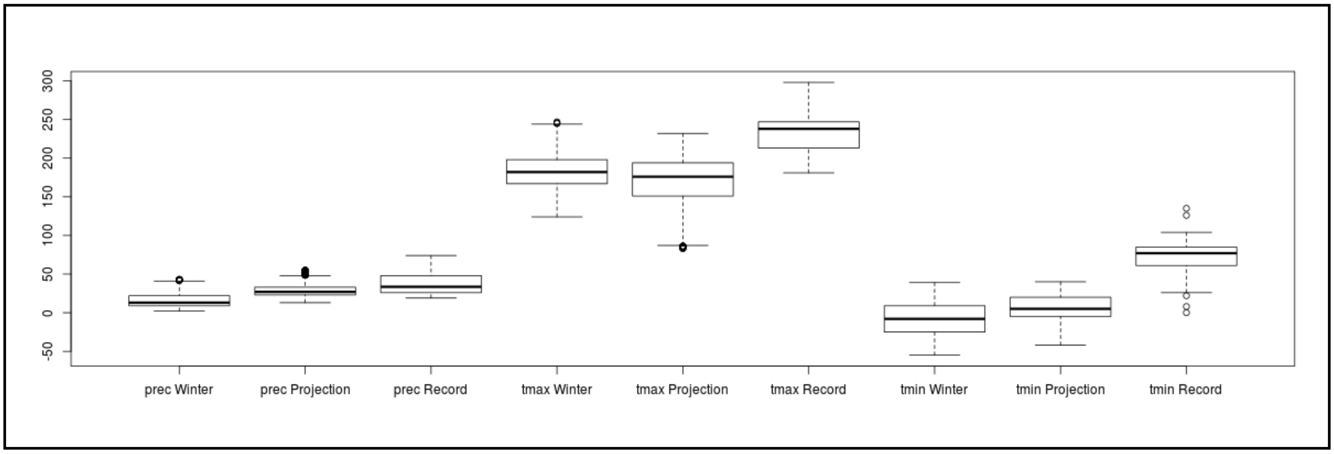

Winter to October

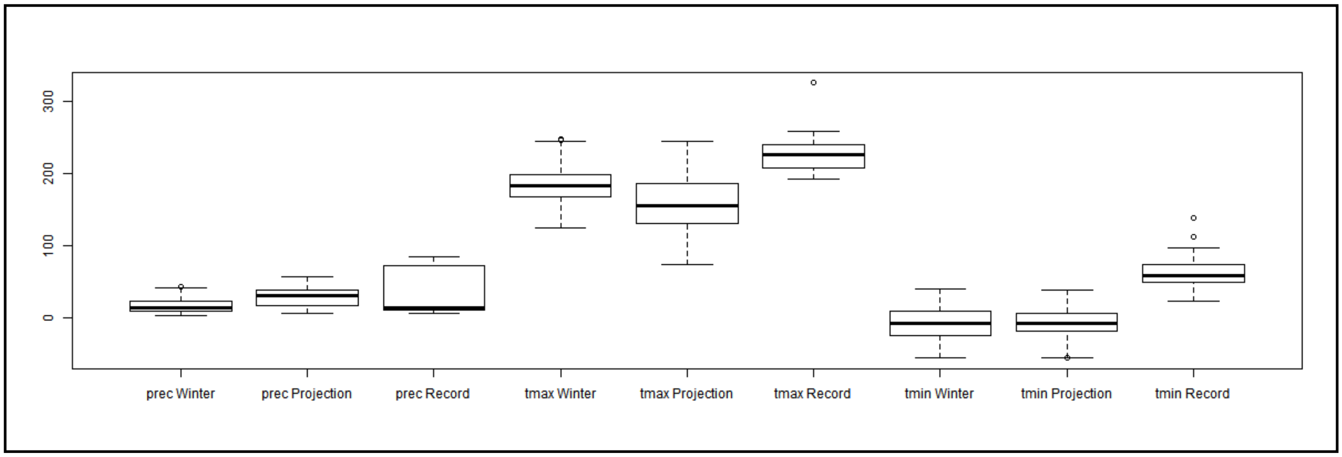

Winter to April

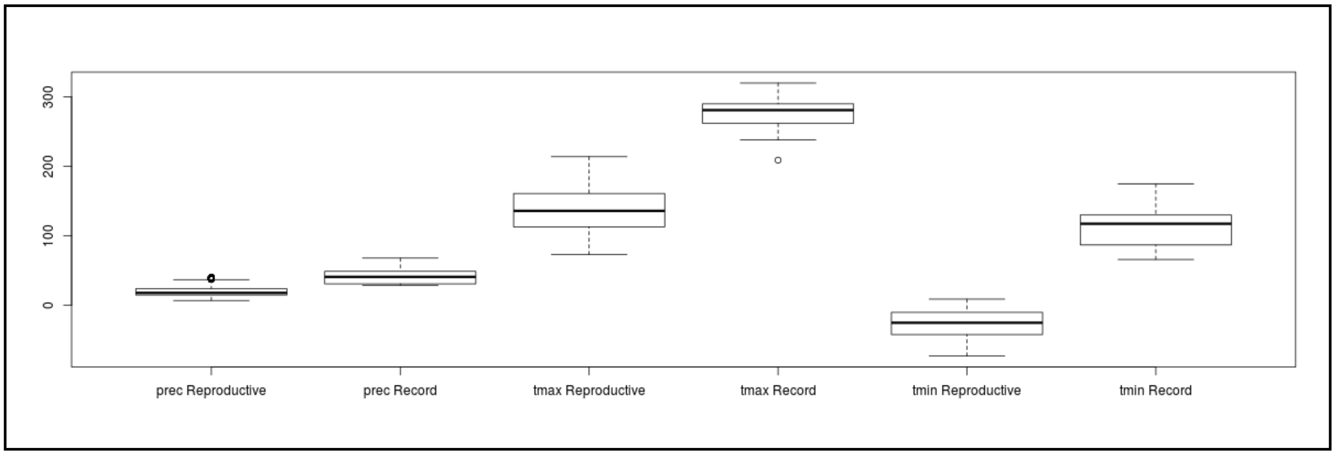

Reproductive to September

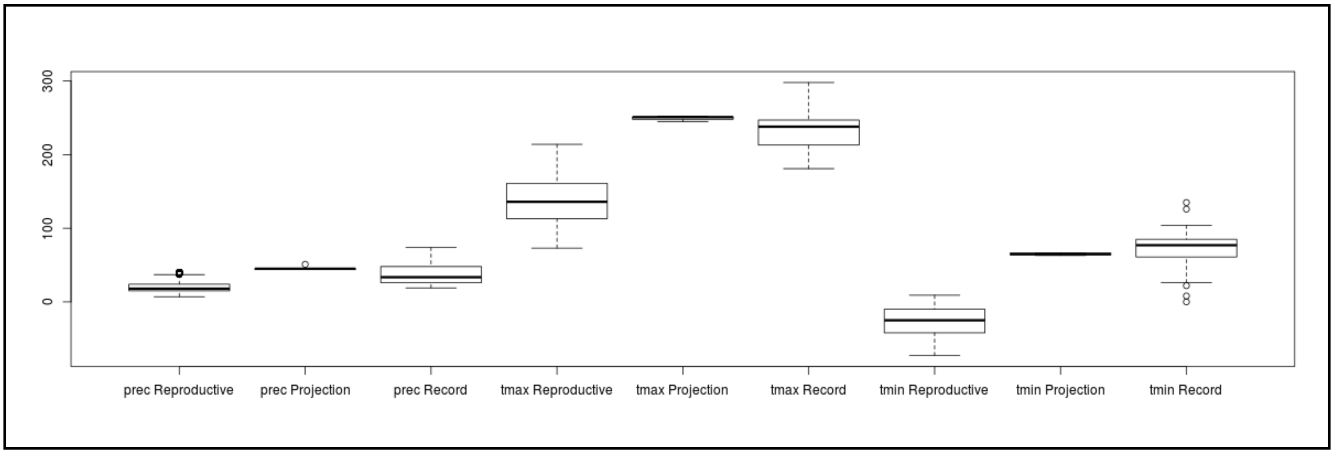

Reproductive to October

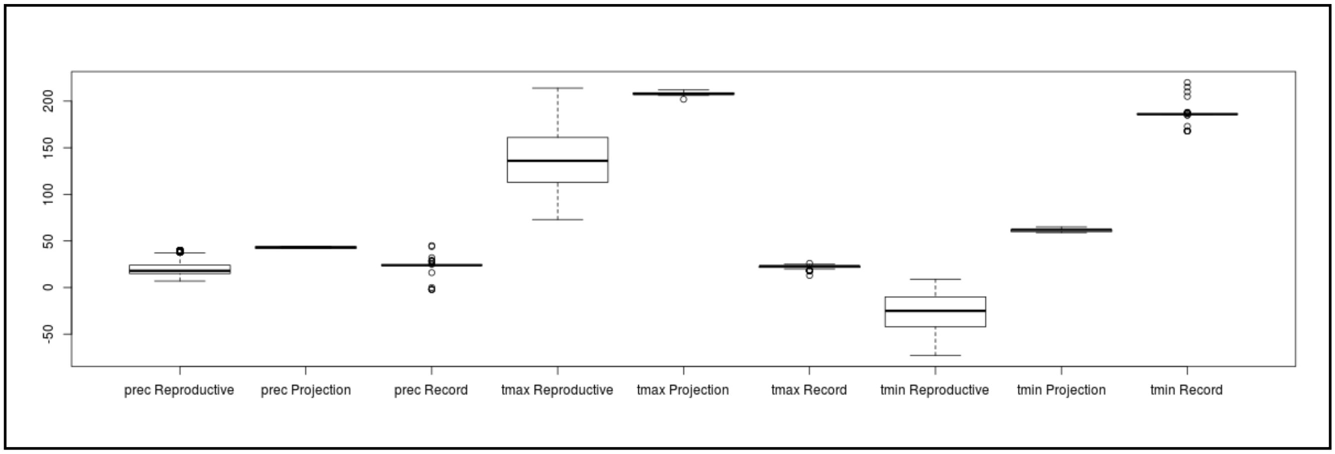

Reproductive to November

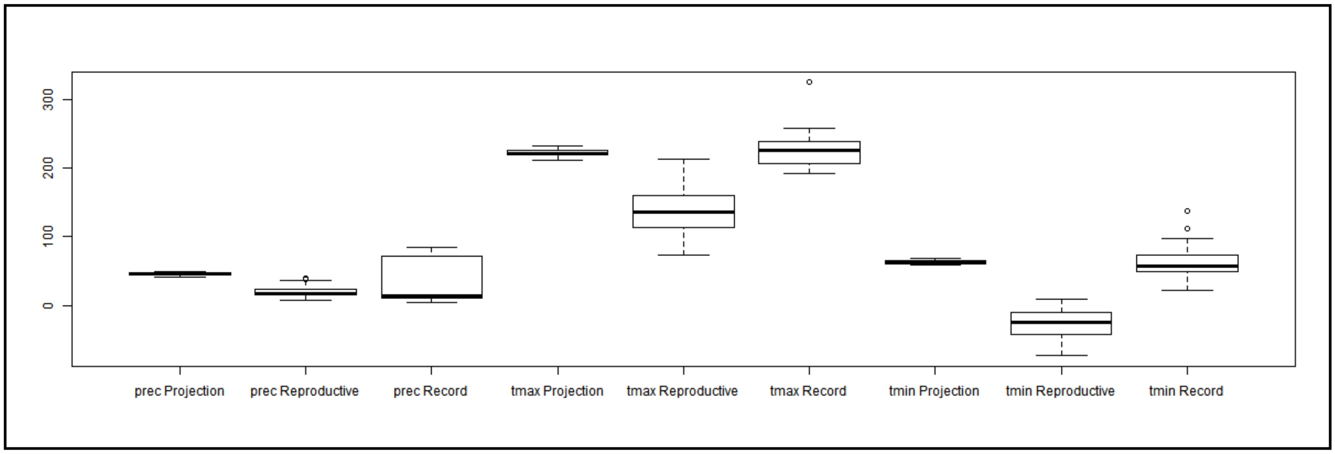

Reproductive to April
